# Improving photodynamic therapy efficacy in bladder cancer using polymer micelle-encapsulated pheophorbide *a*

**DOI:** 10.1016/j.tranon.2026.102687

**Published:** 2026-02-06

**Authors:** Maxime Labroy, Stéphane Chabaud, Maud Durand, Isabelle Fourquaux, Stéphane Bolduc, François Bordeleau, Laure Gibot

**Affiliations:** a Université de Toulouse, CNRS UMR 5623, Laboratoire Softmat, 31062 Toulouse, France; b Centre de Recherche du CHU de Québec – Université Laval, Québec, QC, G1J 0Z4, Canada; c Centre de Recherche en Organogénèse Expérimentale de L’université Laval/LOEX, Université Laval, Québec, QC, G1J 0Z4, Canada; d Centre de Recherche sur le Cancer, Université Laval, Québec, QC, G1V 0E8, Canada; e Centre de Microscopie Electronique Appliquée à la Biologie (CMEAB), Faculté de Médecine Toulouse Rangueil, Université de Toulouse, 31062 Toulouse, France; f Department of Surgery, Faculty of Medicine, Laval University, Québec, QC, G1V 0A6, Canada; g Department of Molecular Biology, Medical Biochemistry and Pathology, Faculty of Medicine, Laval University, Québec, QC, G1V 0A6, Canada

**Keywords:** Photodynamic therapy (PDT), 3D model, Phototoxicity, Microbladder, Spheroids

## Abstract

**Background:**

Photodynamic diagnosis (PDD) is widely used in bladder cancer management, enabling fluorescence-guided detection of lesions through intravesical administration of photosensitizers such as hexaminolevulinate (Cysview®/Hexvix®). Building on this clinical framework, we explored photodynamic therapy (PDT) using pheophorbide *a* (pheo), a chlorophyll-based photosensitizer, encapsulated in self-assembled poly(ethylene oxide)-block-poly(*ε*-caprolactone) (PEO_5000_-PCL_4000_) micelles.

**Results:**

*In vitro* assays were performed on human bladder cancer cell lines, namely the grade 3 invasive T24 and the grade 1 SW780, in both 2D monolayers and 3D spheroid cultures. In 2D, encapsulated pheo showed higher phototoxicity (IC₅₀: 129 nM T24, 156 nM SW780), while free pheo exhibited negligible effects, preventing IC₅₀ determination. Two-photon microscopy confirmed that encapsulation markedly enhanced pheo penetration, especially in T24. SW780 spheroids exhibited tight epithelial features and low permeability, forming characteristic microbladder-like vesicles. PDT reduced viability in both T24 and SW780 3D models, with a significant advantage for encapsulated pheo at day 6 post-treatment in T24 spheroids. Preliminary exploration in human tissue-engineered bladder tumor substitutes demonstrated the feasibility for PDT assessment in complex 3D environments, warranting further study.

**Conclusions:**

These findings support polymer nanocarrier-mediated pheo delivery as a promising therapeutic approach and pave the way for integrated diagnostic–therapeutic strategies using existing intravesical platforms.

## Highlights

• Micelle encapsulation enhances pheophorbide *a* photodynamic efficacy in bladder cancer.
• PEO–PCL micelles improve photosensitizer uptake and tissue penetration.
• PDT efficacy is shown in both 2D cultures and 3D bladder cancer spheroids.
• The tissue-engineered 3D model mimics human tumor bladder tissue and supports translational PDT development.
• Results enable dual diagnostic–therapeutic use *via* existing cystoscopy tools.

## Graphical abstract

**Figure:**
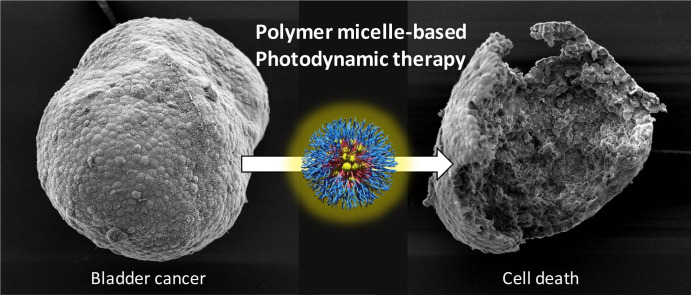

## Introduction

Bladder cancer (BC) is one of the most common cancers globally. According to Global Cancer Observatory (GLOBOCAN) 2022 data, BC is the 9th most frequently diagnosed cancer in the world, with men being about three to four times more likely to be diagnosed than women [1]. The survival rates for BC vary significantly depending on the stage at diagnosis with early-stage disease BC displaying a relatively high survival rate, while advanced-stage disease has a poorer prognosis. Although BC is often diagnosed at an early stage, recurrence and progression remain frequent, leading to prolonged treatment courses [2]. While recent therapeutic advances have improved survival, the cumulative treatment burden, particularly with novel agents, remains poorly characterized regarding patient-reported outcomes and quality of life, rendering BC both complex and costly to manage [3]. Moreover, despite progress in available treatments, striking an optimal balance between efficacy, safety, and cost-effectiveness continues to challenge many healthcare systems [4].

Standard therapeutic strategies for non-muscle-invasive bladder cancer (NMIBC) include transurethral resection of the bladder tumor (TURBT), typically followed by intravesical instillation of Bacillus Calmette-Guérin (BCG) or chemotherapeutic agents such as mitomycin C or gemcitabine [5,6].

However, BCG treatment is associated with variable patient response rates and, in some cases, intolerance[7,8]. Furthermore, recurrent shortages of BCG underscore persistent and clinically significant unmet therapeutic needs. In BCG-unresponsive cases, radical cystectomy remains the gold standard despite its association with significant morbidity and patient reluctance. Consequently, there is growing interest in bladder-sparing alternatives [9].

Photodynamic diagnosis (PDD) is already well integrated into the diagnosis of BC, utilizing specific photosensitizers based on hexyl-aminolevulinic acid ester such as FDA- and EMA- approved Cysview® and Hexvix®. When intravesically instilled, these photosensitizers allow for precise visualization of tumor lesions under the blue light emitted by the cystoscopes (380- to 450-nm wavelength). A systematic review indicated that PDD with 5-aminolevulinic acid (5-ALA) detects more bladder tumor-positive patients, particularly those with carcinoma *in situ*, than conventional white-light cystoscopy [10]. Moreover, more patients present a complete resection and a longer recurrence-free survival when diagnosed with PDD. This PDD technology demonstrates that the essential elements for photodynamic therapy (PDT) are already in place in clinics. PDT involves the use of light-sensitive compounds that, when exposed to specific wavelengths of light, produce reactive oxygen species that can destroy cancer cells. Therefore, the photosensitizers and equipment used for PDD can be readily adapted to PDT, where light not only helps in diagnosis but also in treating tumors. Since the first report of PDT in NMIBC by Benson et al. in 1983 [11], several clinical studies have been conducted and recently meta-analyzed [12]. Clinical results indicate that both therapeutic and adjuvant PDT present satisfactory safety and efficacy for NMIBC, including these cases that are resistant to the standard of care. In a similar way, results in animal models highlight the potential use of PDT to reduce local recurrence in BC [13]. Thus PDT can be used as an alternative to current methods like TURBT, particularly for patients who are not good candidates for radical surgery.

Pheophorbide *a*, a chlorophyll-derived photosensitizer belonging to the porphyrin family [14], was used as a model compound for PDT. Pheophorbide *a* absorbs light both in the blue region (≈ 400–410 nm) and in the red region (≈ 665–670 nm), with its blue absorption compatible with the excitation wavelengths used in clinical blue-light cystoscopy systems (380–450 nm). Like many photosensitizers with extended aromatic structures, it is highly hydrophobic, which limits its solubility in aqueous media and promotes aggregation. To overcome these limitations and preserve its photophysical properties, particularly its singlet oxygen quantum yield, encapsulation strategies are necessary to ensure optimal dispersion, reduce aggregation, and enhance therapeutic performance. In this study, polymer-based nanocarriers were selected to improve the solubility and delivery of pheophorbide *a*. Specifically, self-assembled micelles formed from the amphiphilic block copolymer poly(ethylene oxide)-block-poly(*ε*-caprolactone) (PEO-PCL) were employed, due to their well-known biocompatibility and biodegradability [15]. These micelles provide an hydrophilic crown and a suitable hydrophobic core for the encapsulation of pheophorbide *a*, helping to prevent aggregation, maintain photophysical properties, and improve formulation stability under physiological conditions [16].

This study directly compares free *versus* encapsulated photosensitizer in 2D monolayers, 3D tumor spheroid and tissue-engineered models of two BC cell lines, namely the grade 3 invasive T24 and the grade 1 non-invasive SW780. The aim was to evaluate penetration, phototoxic efficacy, and therefore the potential for clinical translation through intravesical instillation, a delivery route compatible with current clinical practice.

## Material and methods

### Chemicals

Poly (ethylene oxide)-*block*-poly (ε-caprolactone) (PEO-PCL) 5000–4000 was purchased from Polymer Source (Polymer Source, Inc., Dorval, Canada). Pheophorbide *a* (Pheo) was purchased from Frontier Scientific (Logan, Utah, USA). Acetone and phosphate buffer saline (PBS) were purchased from Sigma Aldzrich (Merck, Darmstadt, Germany). Ultrapure water at 18,2 MΩ.cm resistivity was obtained with an ELGA Purelab Flex system (ELGA LabWater, High Wycombe, UK).

### Preparation and characterization of block-copolymer micelles

PEO-PCL block copolymer micelles were prepared and characterized as described previously [17]. Briefly, 20 mg of PEO-PCL copolymers were dispersed in 400 μL of acetone, and this solution was slowly added at room temperature to 5 mL of water (filtered on 0.22 µm RC filters) while stirring at 800 ± 50 rpm (Variomag Poly magnetic stirrer). Acetone was left to evaporate for 48 h. For Pheo-loaded PEO-PCL micelles (pheo-PEO-PCL), a known amount of a stock solution (0.5 mg.mL^-1^) of the photosensitizer in acetone was added to the polymer solution prior to its addition to the water solution. The molar ratio between the photosensitizer and PEO-PCL polymer was kept constant at 1/30 mol/mol to ensure complete encapsulation. The physico-chemical characterization of these systems has been largely described previously [[16], [17], [18], [19], [20]]. The hydrodynamic sizes of the micelles were determined by Dynamic Light Scattering (DLS) on a Malvern Zetasizer instrument, using the general-purpose, non-negative least squares routine for data processing. Empty PEO-PCL micelles and Pheo-loaded PEO-PCL micelles had respective number mean hydrodynamic diameters of dh = 18 ± 3.3 nm and 14 ± 1.1 nm [20]. Polymer micelle shape was observed by transmission electron microscopy as described previously [17,21,20], which confirmed that the copolymers formed spherical nano-objects.

### Stability of PEO–PCL micelles in artificial urine

Artificial urine was prepared according to a published protocol [22]. Its composition was as follows: Na₂SO₄ (11.96 mM), uric acid C₅H₄N₄O₃ (1.49 mM), Na₃C₆H₅O₇·2H₂O (2.45 mM), creatinine C₄H₇N₃O (7.8 mM), urea CH₄N₂O (249.75 mM), KCl (30.95 mM), NaCl (30.05 mM), CaCl₂ (1.66 mM), NH₄Cl (23.67 mM), MgSO₄·7H₂O (4.39 mM), NaH₂PO₄·2H₂O (18.67 mM), and Na₂HPO₄ (5.64 mM). All chemicals were purchased from Sigma Aldrich (Merck, Darmstadt, Germany) and used without further purification. The components were dissolved in 20 mL ultrapure water under magnetic stirring (250–500 rpm) and filtered through a 0.22 µm regenerated cellulose filter. Empty PEO–PCL micelles were then incubated in artificial urine at 37 °C. Hydrodynamic diameters were measured at selected timepoints by dynamic light scattering (DLS) at 25 °C using a Malvern Zetasizer and processed using the general-purpose non-negative least squares algorithm.

### Cell culture

Human BC cell lines T24 (ATCC® HTB-4™, derived from a grade 3 transitional cell carcinoma of an 81-year-old woman) and SW780 (ATCC® CRL-2169™, grade 1 transitional cell carcinoma from an 80-year-old woman) were purchased in 2022 from ATCC and cultured in Dulbecco’s Modified Eagles Medium containing GlutaMAX supplemented with 10 % of heat-inactivated fetal calf serum, 100 U.mL^-1^ of penicillin, and 100 µg.mL^-1^ of streptomycin at 37 °C in a humidified atmosphere at 5 % CO_2_ (Panasonic, MCO-170AiCUVH). Cell culture media were changed three times a week, and cells were tested negative for mycoplasma using the MycoAlert mycoplasma detection kit (Lonza, Verviers, Belgium) throughout the experiments.

### Cell viability assessment after photodynamic therapy on monolayers

The day before the experiments, 5000 T24 or SW780 cells were seeded in flat-bottom 96-well plates. On the day of treatment, cells were incubated for 30 min at 37 °C with increasing concentrations of empty PEO-PCL micelles, free pheophorbide (pheo), or pheo-loaded PEO-PCL micelles. A spotlight equipped with a band-pass filter (λ > 400 nm) and protected by a glass slide was positioned 4 cm above the samples. Cells were then light-irradiated for a total of 4 min (2 min on, 2 min off to avoid thermal effects, followed by 2 min on), as previously described [17,19,21,23,24]. Each well received a total fluence of 8.2 J/cm². This illumination protocol corresponded to a fluence rate of approximately 34 mW/cm². Plates were transferred to an Incucyte S3 live-cell imaging system (Sartorius), and images were acquired every 6 h at 10 × magnification for a period of 72 h. Cell confluence over time was quantified using the associated Incucyte analysis software. At least one experiment (N) with 6 biological replicates (n) each time were led.

### Migration assay after photodynamic therapy on monolayers

The migratory capacity of T24 and SW780 BC cells was assessed using a scratch-wound assay. On the morning preceding the experiment, 40,000 cells were seeded per well in ImageLock 96-well plates (Sartorius). Approximately 8 h later, cells were serum-starved overnight in FBS-free medium to minimize the contribution of proliferation and isolate the migratory component in wound healing. On the day of the assay, a standardized wound (750 µm in width) was generated in each well using the WoundMaker device (Sartorius). Detached cells were removed by washing, and photodynamic therapy (PDT) treatments were performed in 100 µl of cell culture medium as described above. Plates were subsequently placed in the IncuCyte S3 Live-Cell Analysis System (Sartorius), and phase-contrast images were acquired every hour for 24 h at 10 × magnification. Wound closure dynamics were quantified using the integrated Scratch Wound module.

### Photodynamic therapy of 3D tumor spheroids

T24 and SW780 tumor spheroids were produced by the non-adherent technique as previously described [25,26] using ultra-low attachment 96-well plates (Costar #7007, Fisher Scientific, Illkirch, France). Briefly, 5000 cells in suspension were seeded in 300 μL of cell culture medium in each round-bottom well. Spheroids were cultivated for 5 days at 37 °C in a humidified atmosphere containing 5 % CO_2_ before experiments were performed. On the day of the treatment, T24 and SW780 spheroids were incubated with free pheophorbide or pheo-loaded PEO-PCL micelles for 30 min at 37 °C in 300 µL of cell culture medium. Concentration of pheophorbide was set at 3 µM as previously described for PDT in 3D spheroids [16,23]. A spotlight protected by a glass slide (band pass filter λ > 400 nm) was placed 4 cm above the samples. Spheroids were light-irradiated for a total of 8 min (4 min light on, 2 min light off in order to avoid heating and then 4 min light on). Each was received a total fluence 16.4 J/cm². This illumination protocol corresponded to a fluence rate of approximately 34 mW/cm². Three and six days after PDT treatment, spheroid’s cell viability was assessed by quantifying intracellular ATP content using CellTiterGlo3D luminescent kit (#G9682, Promega, Charbonnières-les-Bains, France) according to the manufacturer protocol’s and as previously described [16]. Briefly, cell culture medium was removed in order so that only 50 µl of supernatant were let with the spheroid in the well. The same volume of reagent was added and incubated under vigorous shaking at room temperature for 30 min, protected from light. After spheroid lysis, the whole content of each well (i.e. 100 µl) was transferred into a white-96-well plates and luminescence was read on plate reader Synergy H1 (BioTek Instruments, Winooski, VT, USA).

### Scanning and transmission electron microscopy

For electron microscopy, cells were grown the day prior to the experiments on 12 mm- gelatin-coated round glass coverslips in a 24-well plate, or grown under spheroid configuration for 5 days before being fixed. One day after seeding, 2D cells were fixed in 0.1 M Sorensen phosphate buffer (pH 7.4) containing 2 % of glutaraldehyde for 24 h at 4 °C and processed as classically described [21,27]. Observations were carried out using a Hitachi HT7700 transmission electron microscope and an FEI Quanta 250 FEG scanning electron microscope.

### Pheophorbide *a* penetration in 3D spheroids assessed by two-photon microscopy

To evaluate the spatial distribution of pheophorbide *a* (red fluorescence) in 3D tumor spheroids, 5-day-old T24 or SW780 spheroids were incubated with empty PEO-PCL micelles, or 3 µM free pheo or pheo-loaded PEO-PCL micelles for 30 min at 37 °C. Fresh samples were then imaged using a Zeiss 7MP two-photon microscope (Carl Zeiss, Jena, Germany) equipped with a Chameleon Ultra II Ti:Sapphire laser (Coherent Inc.), tuned to an excitation wavelength of 800 nm. Emission was collected through a 640–710 nm dichroic filter, matching the fluorescence emission window of pheophorbide *a* (emission peak ∼680 nm). Image stacks covering a volume of 420 × 420 × 120 μm were acquired. Post-processing and image visualization were performed using ImageJ software (NIH, Bethesda, MD, USA). Two-photon excitation was used exclusively for deep imaging of spheroids and not for photosensitizer activation.

### Production of human BC substitutes and photodynamic treatment

All procedures involving patients were conducted according to the Declaration of Helsinki and were approved by the Research Ethics Committee of CHU de Québec-Université Laval (2012–1341). Donors’ consent for tissue harvesting was obtained for each specimen (DR-002–1190). Experimental procedures were performed in compliance with the CHU de Québec guidelines. Human bladder tissue substitutes were produced using the self-assembly technique [28], as previously described [29,30], with minor adaptations. Briefly, normal human bladder fibroblasts and dermal fibroblasts were co-seeded at a density of 3 × 10⁴ cells.cm^-^² (80:20 ratio) in 6-well plates and cultured for 21 days in medium supplemented with 50 μg/mL ascorbic acid to stimulate extracellular matrix production [31]. Once fibroblast-derived stromal sheets were formed, three sheets were stacked to constitute the stromal compartment. Urothelial cells were then seeded at 3 × 10⁴ cells.cm^-^² on top of the stacked stroma and maintained at the air–liquid interface for an additional 21 days to allow terminal urothelial differentiation. To generate BC substitutes, tumor spheroids composed of either T24 or SW780 cells were produced separately and deposited on the urothelial surface of the reconstructed tissues (five spheroids per tissue). After 3 h of incubation at 37 °C to allow adhesion, tissues were returned to the air–liquid interface and cultured for 7 days to permit spheroid integration (Figure S1). For photodynamic therapy protocol, 200 µL of culture medium containing 3 µM of free or PEO-PCL-encapsulated pheophorbide *a* were gently applied onto the surface of the tissues and incubated for 2 h at 37 °C in the dark. Samples were then illuminated as described above for spheroid treatment (4 min light on, 2 min light off to avoid heating and then 4 min light on). Tissue viability was assessed at 48 and 72 h post-PDT by incubating the bladder substitutes for 30 minutes at 37 °C, protected from light, with PrestoBlue reagent (ThermoFisher Scientific, Illkirch, France) diluted 1:10 in PBS, according to the manufacturer's instructions. Absorbance of the supernatant was measured at 570 and 600 nm using a plate reader. As the assay is non-destructive, samples were returned to culture in fresh medium until the final 72-hour time point. Tissues were then fixed in 4 % paraformaldehyde 72 h post-PDT, embedded in paraffin, and processed for Masson's trichrome staining.

### Statistical analyses

At least six biological replicates and up to 39 (n) were produced and analyzed for each independent experiment (N) in monolayer or spheroid experiments. Data analyses were performed using GraphPad Prism 10.4.1 (GraphPad Software, Inc., La Jolla, *CA*, USA), and independent biological replicates were plotted and expressed as mean ± SEM (standard error of the mean). Statistical comparisons were performed using ordinary one-way analysis of variance (ANOVA), followed by Tukey’s post hoc test to compare all conditions against each other. Statistical significance symbols displayed directly above each column indicate comparisons to their respective 0 µM control (either non-irradiated or irradiated). For 3D BC tissue viability assays at 48 h and 72 h post-photodynamic treatment (PDT), absorbance values were expressed as a percentage of the control (untreated) condition at the corresponding timepoint. Due to insufficient replicates in the control group (*n* = 1), statistical comparisons were restricted to the Pheo (*n* = 5) and Pheo-PEO-PCL (*n* = 4) conditions using unpaired two-tailed Student’s *t*-tests. Normality of the data was confirmed using the Shapiro–Wilk test. Overall statistical significance was set at *p < 0.05, **p < 0.01, ***p < 0.001, and ****p < 0.0001, ns = non-significant.

## Results and discussion

### Relative stability of empty PEO-PCL micelles in artificial urine

In order to ensure the relevance of biological experiments, the stability of the empty micelles was verified in artificial urine [22]. The artificial urine was produced the day before the experiments, and filtered and used on the day of experiment. Its measured pH was 6.26, which is similar to normal urine [22]. The stock solution of poly (ethylene oxide)-block-poly (ε-caprolactone) micelles (PEO-PCL) was diluted 1/9 to achieve the same concentration as in the *in vitro* experiments (polymer concentration similar to 1.65 µM pheo). The stability of the same solution was measured by DLS at different time intervals following incubation at 37 °C. The results are shown in Table 1. The dilution of the PEOPCL micelles in artificial urine seems to promote the formation of large aggregates, which is visible from the increase of the polydispersity index from 0.8 for the stock solution to 1.0 for the 0 min dilution in artificial urine. Furthermore, during the DLS analysis replicates of diluted PEOPCL micelles in urine (0 min and 12 min), an increase in size of the population could be observed. After 2 h at 37 °C in artificial urine, the micelles seem to have further evolved with a decrease of the polydispersity index but remaining in the same range of number-averaged diameter as at the beginning of the experiment. Finally, the observed derived count rate when micelles were diluted in artificial urine was reasonably stable, indicating that the amount of micelles remains similar. This is coherent with literature showing that urea (main component of this artificial urine with 249.75 mM) interact preferentially with poly(ethylene oxide) (PEO) in the case of with poly(ethylene oxide)−poly(propylene oxide)−poly(ethylene oxide) (PEO−PPO−PEO) triblock copolymers [32]. These physicochemical results clearly warrant further investigation in a more comprehensive study, for example with a FRET experiment between pheo and a DiO-type fluorescent probe in the hydrophobic part of the micelles to ensure the integrity of the objects in urine, or their dissociation kinetics, but this is clearly beyond the scope of this study.

**Table 1:** **Stability of empty PEO–PCL micelles in artificial urine.** Hydrodynamic diameters of empty poly(ethylene oxide)-block-poly (ε-caprolactone) (PEO-PCL) micelles measured by DLS after incubation in artificial urine (dilution 1:9). The stock micelle solution (in water) was analyzed first, followed by measurements at 0 and 12 min (25 °C) and 120 min (37 °C) post-dilution. Size corresponds to the number-averaged diameter (nm); PDI denotes the polydispersity index; derived count rate is expressed in kilocounts (kcounts).

Sample	Time (min)	Size (nm)	PDI	kcounts
PEOPCL stock solution (water)	0 min	26	0.8	17,900
PEOPCL (urine)	0 min	24	1.0	3530
PEOPCL (urine)	12 min (at 25 °C)	28	1.0	2200
PEOPCL (urine)	120 min (at 37 °C)	32	0.5	2600

### Relative stability of empty PEO–PCL micelles in artificial urine

To ensure the relevance of the biological experiments, the stability of empty micelles was assessed in artificial urine [22]. Artificial urine was prepared the day before the experiments and filtered immediately prior to use. Its measured pH was 6.26, consistent with physiological urine values [22]. The stock solution of poly(ethylene oxide)-block-poly(ε-caprolactone) (PEO–PCL) micelles was diluted 1:9 to match the polymer concentration used in the *in vitro* assays (corresponding to that of 1.65 µM Pheo). The stability of this diluted micelle suspension was evaluated by DLS at several timepoints following incubation at 37 °C. Results are presented in Table 1. Dilution of PEO–PCL micelles into artificial urine appeared to promote the formation of larger aggregates, as suggested by the increase in polydispersity index (PDI) from 0.8 in the stock solution to 1.0 immediately after dilution (0 min). During replicate DLS measurements at 0 and 12 min, a progressive shift toward larger apparent diameters was consistently observed. After 2 h of incubation at 37 °C, the micelles displayed a slightly reduced PDI while maintaining number-averaged diameters in the same overall range as at the start of the experiment. Importantly, the derived count rate remained relatively stable across measurements for diluted micelles in urine, indicating that the overall amount of scattering objects in solution was maintained. This observation is consistent with reports showing preferential interactions between urea, the major component of artificial urine (249.75 mM), and poly(ethylene oxide) (PEO) segments in amphiphilic block copolymers such as PEO–PPO–PEO triblocks [32]. Although these physicochemical results suggest that PEO–PCL micelles undergo some degree of structural rearrangement upon dilution in artificial urine, they do not indicate a loss of micellar material. A more detailed investigation, for example using FRET between DiI and DiO probes inserted in the hydrophobic core to monitor micelle integrity or dissociation kinetics [17], would be required to fully resolve these mechanisms. However, such analyses fall beyond the scope of the present study.

### Morphological assessment in monolayer reveals distinctive features in T24 and SW780 BC cell lines

**Fig. 1:**
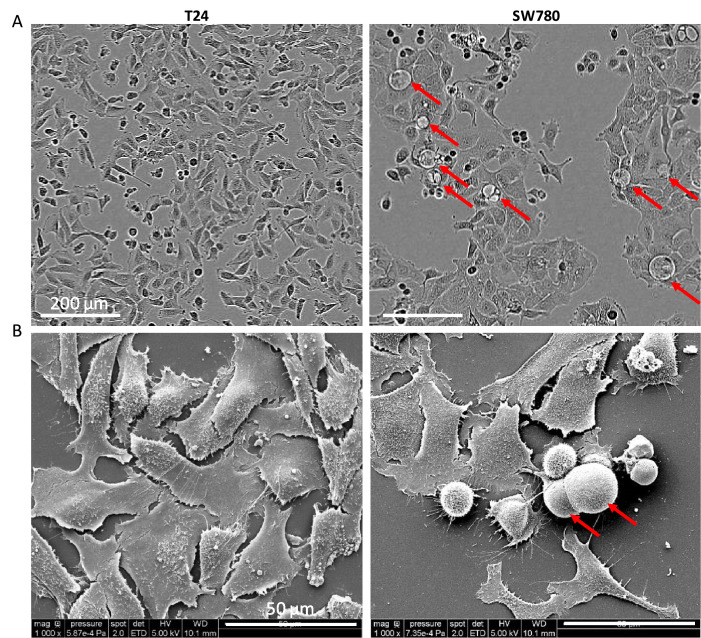
SW780 bladder cancer cells produce floating microbladder-like vesicles (luminal cavities characteristic of urothelial differentiation) in monolayer culture. A. Phase-contrast microscopy of T24 and SW780 bladder cancer cells. B. Scanning electron microscopy (SEM) images showing cell surface morphology. Red arrows indicate floating cyst-like vesicles (microbladder-like, luminal cavities characteristic of urothelial differentiation) produced by the SW780 cell line. Images are representative of the overall cell population.

Both phase-contrast microscopy and scanning electron microscopy (SEM) analyses demonstrated that the two BC cell lines displayed characteristic epithelial morphology (Fig. 1). Specifically, the cells exhibited a polygonal shape with clearly defined cell boundaries, prominent cell-cell junctions, and a closely packed arrangement typical of epithelial tissue architecture. The presence of cyst-like structures, interchangeably referred to as microbladders (luminal cavities characteristic of urothelial differentiation) throughout the manuscript, was observed in monolayer cultures of SW780 cells as floating vesicles located extracellularly, emerging from cell plasma membranes. In contrast, T24 cells, classified as grade 3, did not exhibit this behavior. T24 cells were derived from high-grade transitional cell carcinoma (grade 3), and according to the literature, they are aggressive, exhibiting a high proliferative rate, with recurrence or progression to muscle invasion and metastasize at a greater frequency [33,34]. Conversely, SW780 cells were derived from a low-grade (grade 1) transitional cell carcinoma and exhibited characteristics similar to normal urothelial cells. Notably, they were well-differentiated and showed slower proliferation, consistent with the typical behavior of low-grade BC [33]. Transmission electron microscopy (TEM) analyses of these monolayers (Figure S2) did not reveal the presence of intracellular vesicles, *i.e.*cyst-like structures, in either T24 or SW780 cells. A more detailed characterization of the cyst-like structures formed by SW780 cells in 3D culture is shown in Figs. 3B and 3C. Within spheroids, these cells organized into epithelial-like structures with well-defined lumens, highlighting their tendency to adopt a cystic architecture in a three-dimensional epithelial context.

### Encapsulation of photosensitizer in PEO–PCL micelles enhances phototoxicity in 2D BC cells

**Fig. 2:**
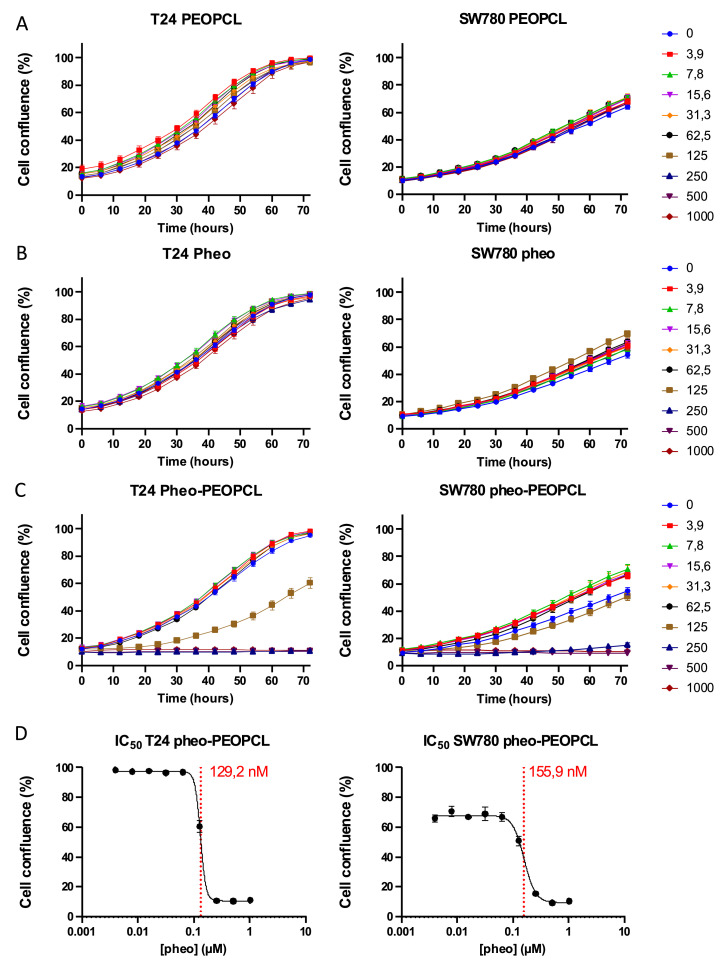
**Encapsulation of pheophorbide *a* in PEO-PCL micelles enhances PDT-induced cytotoxicity in 2D high-grade (T24) and low-grade (SW780) bladder cancer cell cultures.** A. Cells were treated with empty poly (ethylene oxide)-block-poly (ε-caprolactone) PEO-PCL micelles (PEOPCL) at polymer concentrations equivalent to those used in pheo-loaded micelles, based on the 1:30 drug-to-polymer weight ratio. B. Cells were exposed to increasing concentrations of free pheophorbide a (Pheo) (nM). C. Cells were treated with increasing concentrations of Pheo encapsulated in PEO-PCL micelles (pheo–PEOPCL) (nM). D. Half-maximal inhibitory concentration (IC₅₀) determination for the Pheo–PEOPCL condition. Cell confluence was monitored by videomicroscopy for 72 h post-PDT (*N* > 1; *n* = 6).

To assess the phototoxic potential of pheophorbide *a* in BC cells, the effects of free *versus*micelle-encapsulated Pheo were compared in standard 2D monolayer cultures of T24 (grade 3) and SW780 (grade 1) cells, using real-time videomicroscopy to monitor cell confluence for 72 h post-PDT (Fig. 2). Control experiments using empty PEO-PCL micelles at polymer concentrations equivalent to those in drug-loaded formulations (based on a 1:30 drug-to-polymer ratio) showed no phototoxicity in either cell line (Figure 2A). Treatment with increasing concentrations of free Pheo (0–1 µM) followed by PDT showed negligible phototoxicity (Fig. 2B) and no quantifiable IC₅₀ under the same conditions (Figure 2D). In contrast, Pheo encapsulated in PEO-PCL micelles (Pheo-PEO-PCL) produced a concentration-dependent reduction in confluence in both cell lines (Figure 2C), with a clear photodynamic effect detectable even at low nanomolar doses. Although this readout does not distinguish between cell death and cytostatic effects, the near-complete loss of confluence at the highest concentration suggests that the encapsulated Pheo triggered robust cell killing rather than merely inhibiting proliferation. IC₅₀ values derived from these kinetic curves confirmed the superior efficacy of Pheo-PEO-PCL formulation, with IC₅₀ values in a similar range for both cell lines, namely 0.129 µM for T24 (R² = 0.992) and 0.156 µM for SW780 cells (R² = 0.912) (Figure 2D). These findings were consistent with our previous studies in head and neck (FaDU) [23], melanoma (A375) and colorectal (HCT-116) cancer cell lines where Pheo encapsulation in PEO-PCL micelles similarly enhanced PDT efficacy, supporting the generalizability of this approach across diverse cell types.

### Encapsulation of photosensitizer in PEO–PCL micelles enhances phototoxicity in 3D bladder tumor spheroids

**Fig. 3:**
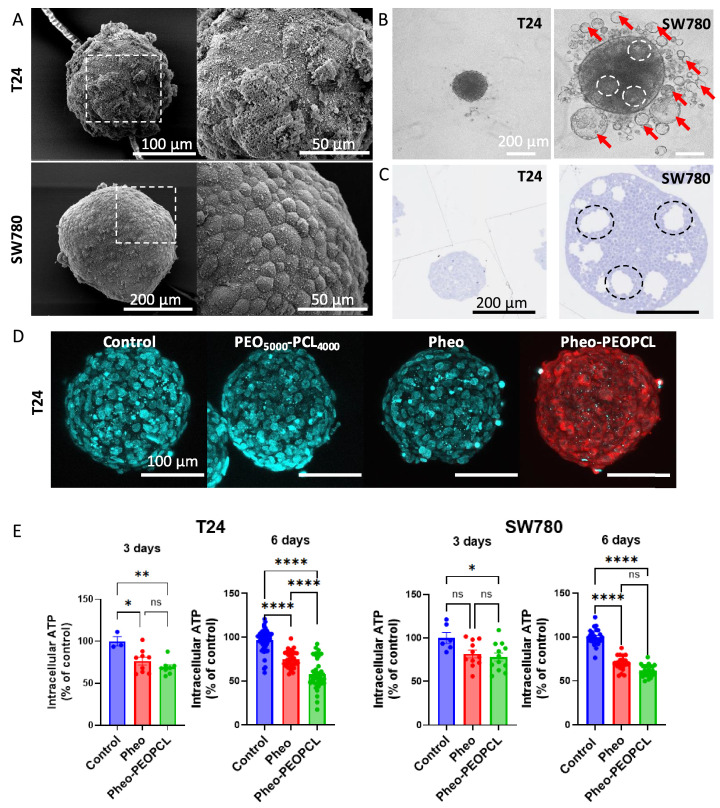
**Encapsulation of pheophorbide *a* enhances PDT efficacy in 3D tumor spheroids.** A. Scanning electron microscopy (SEM) images showing the surface morphology of T24 and SW780 spheroids. B. General appearance of bladder tumor spheroids before treatment. Red arrows indicate detached cyst-like vesicles, i.e. “microbladders” (luminal cavities characteristic of urothelial differentiation); dotted circles highlight internal cyst-like structures embedded within the spheroids. C. Counterstained semi-thin transverse sections (500 nm) of spheroids revealing internal cyst-like structures (dotted circles). D. Two-photon microscopy imaging of pheophorbide *a* (Pheo) (1 µM) penetration in T24 spheroids after 30 min incubation at 37 °C either in its free or encapsulated formulation. Poly (ethylene oxide)-block-poly (ε-caprolactone) (PEO_5000_-PCL_4000_) empty micelles; Pheophorbide encapsulated in PEO-PCL micelles (Pheo-PEOPCL). Cyan: nuclei (Hoechst); red: pheophorbide fluorescence. E. Spheroid viability assessed by intracellular ATP quantification at 3- and 6-days post-PDT ([Pheo] = 3 µM). Results include data from 1 to 6 independent experiments (N), with a cumulative number of biological replicates (n) ranging from 6 to 39.

Scanning electron microscopy (SEM) images (Fig. 3A) highlight distinct morphological differences between T24 and SW780 spheroids. SW780 spheroids were approximately twice as large as T24. The surface of T24 spheroids appeared rough and irregular, covered with unidentified debris, whereas SW780 spheroids displayed a smooth texture with tightly packed cells. Fresh spheroids before treatment (Fig. 3B) were larger than in SEM images, as expected due to dehydration effects in SEM preparation. T24 spheroids were highly spherical. In contrast, SW780 spheroids were surrounded by numerous detached cyst-like vesicles (“microbladders”) (luminal cavities characteristic of urothelial differentiation) that were clearly separate from the spheroid surface and fragile enough to be lost during pipetting. Internal cyst-like regions were also visible within SW780 spheroids. 500 nm-semi-thin stained sections (Fig. 3C) confirmed these observations: T24 spheroids were dense and homogeneous, whereas SW780 spheroids contained internal cyst-like vesicles. This complexity aligns with their epithelial nature, and suggests a resemblance to organoids, consistent with their origin from low-grade urothelial carcinoma cells. Furthermore, transmission electron microscopy (TEM) on SW780 spheroids in Figure S3 revealed an additional level of complexity, demonstrating the presence of intracellular cyst-like vesicles within a subset of the SW780 spheroid cell population. Notably, these intracellular vesicles were predominantly found in cells located adjacent to either the luminal spaces of microbladders or the spheroid surface, suggesting spatial heterogeneity in vesicle formation and localization.

Two-photon microscopy imaging of pheophorbide *a* penetration (Fig. 3D) showed strong fluorescence in T24 spheroids incubated with encapsulated pheo, while free pheo fluorescence was barely detectable. Empty micelles showed no fluorescence. Conversely, no pheo fluorescence nor Hoechst nuclear stain penetrated SW780 spheroids, suggesting an impermeability consistent with their urothelial differentiation and low-grade phenotype, as previously observed [26], which precluded imaging of these structures under two-photon microscopy conditions. Although epithelial tightness was not quantified through dedicated assays such as TEER or junctional staining, the complete exclusion of two independent fluorescent tracers, pheophorbide *a* and Hoechst, provides functional evidence of a highly restrictive barrier. This behavior is fully consistent with the well-differentiated urothelial organization reported for SW780 spheroids and commonly associated with limited permeability in low-grade bladder cancer models.

Some free photosensitizers such as hypericin rely on binding to plasma proteins like human serum albumin for improved cellular uptake, which influenced their accumulation in bladder tumor spheroids, *i.e.* T24 spheroids, compared to normal urothelial spheroids [35]. This selective accumulation in tumor cells was not seen with free pheophorbide *a* or mTHPP (meso‑tetra(m-hydroxyphenyl)porphine). In our study, PEO-PCL was used to encapsulate pheophorbide *a*. PEO-PCL micelles exhibit stealth properties due to their hydrophilic corona, reducing protein adsorption [36]. Therefore, the stealth nature of PEO-PCL micelles could improve photosensitizer delivery and penetration by minimizing non-specific protein interactions in the bladder microenvironment. In their study on MGHU3 bladder tumor spheroids, Xiao et al.,. demonstrated that the intra-spheroid distribution of photosensitizers was strongly influenced by their hydrophilic or hydrophobic nature [37]. Interestingly, when certain hydrophobic photosensitizers were encapsulated in liposomes (∼100 nm in diameter), their penetration into spheroids decreased compared to the free form, likely due to limited diffusion of the larger nanocarriers. In contrast, the PEO-PCL micelles used in our study, with a much smaller diameter (∼20 nm), may offer a better compromise between drug protection and tissue penetration, supporting more efficient delivery into the dense structure of bladder tumor spheroids.

Spheroid viability assessed by intracellular ATP measurement (Fig. 3E) demonstrated statistically significant phototoxic effects for both free and encapsulated pheophorbide in T24 spheroids at day 3, with no significant difference between formulations. At day 6, encapsulated pheo induced a significantly larger decrease in T24 spheroid viability, reducing viability from 96.7 ± 1.7 % in the control to 58.2 ± 2.8 % (p < 0.001) (39.8 % reduction), whereas free pheo caused a moderate decrease to 74.9 ± 1.5 % (*p* < 0.001) (22.6 % reduction). For SW780 spheroids, a modest phototoxic effect was observed at day 3 with encapsulated pheo only, and both treatments significantly reduced viability at day 6 without differences between them. These findings indicate greater PDT efficacy in higher-grade T24 spheroids and a delayed response in SW780. In comparison, Vandepitte et al. [38] observed ∼80 % cell death 24 h post-PDT using 30 µM hypericin complexed with PVP (polyvinylpyrrolidone) to improve its solubility, with a similar light fluence (∼18 J/cm²) on T24 spheroids. Our study, employing a tenfold lower concentration (3 µM) of pheophorbide *a* encapsulated in PEO-PCL micelles under comparable illumination, achieved ∼31 % and ∼42 % viability reduction at days 3 and 6 post-PDT, respectively. Despite differences in photosensitizer structure and formulation strategy, the overall levels of phototoxicity are consistent, reinforcing the robustness and translational potential of our approach.

The improved penetration observed with micelle-encapsulated Pheo is consistent with previously reported advantages of drug delivery using amphiphilic block copolymer micelles [39]. Although the benefits of encapsulation are well established, the mechanisms underlying the interaction of such nanocarriers with cellular membranes remain only partially understood. Several studies suggest that PEO–PCL micelles can promote intracellular delivery of hydrophobic cargos through multiple, non-exclusive pathways. These include mass transfer from micelles to the plasma membrane, during which both polymer and cargo may transiently incorporate into the lipid bilayer [16]; direct transfer of Pheo from the micelle corona or core to the membrane as described by Kerdous et al. [40] and Till et al. [18]; and endocytic uptake of intact micelles [41]. The coexistence of these entry routes provides a plausible explanation for the higher intracellular accumulation of Pheo when encapsulated, compared with free Pheo, which relies solely on passive diffusion across the membrane. Thus, our penetration results align with the current understanding that micellar encapsulation enhances the efficiency and multiplicity of delivery pathways for hydrophobic photosensitizers.

Beyond improved cellular entry, additional factors likely contribute to the superior PDT efficacy of Pheo–PEO-PCL micelles. As previously reported [16], encapsulation limits Pheo aggregation and enhances its photophysical performance, including higher fluorescence intensity and singlet-oxygen yield. Micelles also promote faster and greater intracellular accumulation across different cancer cell lines, which translates into stronger photodynamic responses in both monolayer cultures and 3D spheroids. Together, these effects provide a coherent explanation for the improved therapeutic outcomes observed in bladder cancer models.

### PDT with encapsulated pheophorbide *a* impairs wound closure in BC cell monolayers, likely due to cytotoxic effects

**Fig. 4:**
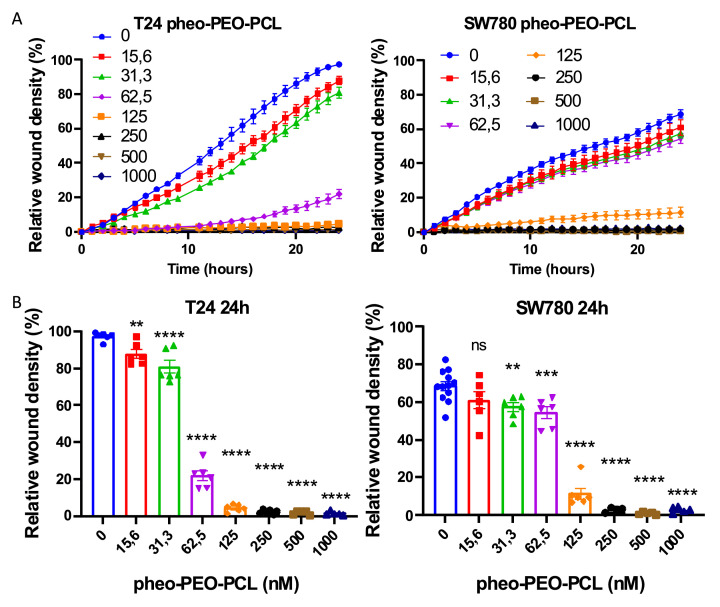
**Reduced wound closure in bladder cancer cell monolayers following photodynamic treatment with encapsulated pheophorbide *a*.** A. Kinetics of wound closure over 24 h post-PDT in T24 (grade 3) and SW780 (grade 1) monolayers, monitored by videomicroscopy. Cells were treated with increasing concentrations of pheophorbide *a* encapsulated in poly (ethylene oxide)-block-poly (ε-caprolactone) PEO-PCL micelles (Pheo-PEO-PCL) (nM). B. Quantification of wound closure ( %) at 24 h post-PDT across the different Pheo-PEO-PCL concentrations. Relative wound density ( %) reflects the proportion of the scratch area repopulated by migrating cells. Data are expressed as mean ± SEM. Statistical analysis was performed using one-way ANOVA followed by Dunnett’s multiple comparisons test *versus*control (0 nM).

In untreated conditions, T24 cells (grade 3) exhibited significantly higher migratory capacity compared to SW780 cells (grade 1), achieving 98 % wound closure within 24 hours *versus*only 69 % for SW780, as measured by live-cell imaging scratch assays (Figure S4, movie S1 and movie S2). A study using chlorophyllin-e6, another chlorophyll-derived photosensitizer such as pheophorbide *a*, reported a marked PDT efficacy on BC cells, including in T24 cells, and a decrease in migration and invasion capacities as assessed by scratch and transwell assays [42]. The conclusion of the authors was that chlorophyllin-e6 PDT may be a powerful tool in the inhibition of cell metastasis. However, such effects should be interpreted cautiously. In our study, the observed inhibition of cell migration post-PDT (Fig. 4) appeared to correlate closely with pheophorbide a-induced cytotoxicity levels (as shown in Fig. 2), suggesting that reduced motility may primarily reflect impaired viability rather than a direct anti-migratory effect. In conditions where the PDT dose approaches or exceeds the IC_50_, it is likely that cells were either undergoing apoptosis or already dead, precluding active migration. Conversely, at sub-lethal doses, a partial inhibition of wound closure may reflect early cytostatic responses or ongoing cell damage. Therefore, while scratch assays provide useful complementary data, they may conflate loss of migratory potential with overall cytotoxicity, especially in the context of potent PDT-induced cell death.

### Towards more physiologically relevant models: preliminary evaluation of PDT efficacy in a 3D human bladder tissue model

**Fig. 5:**
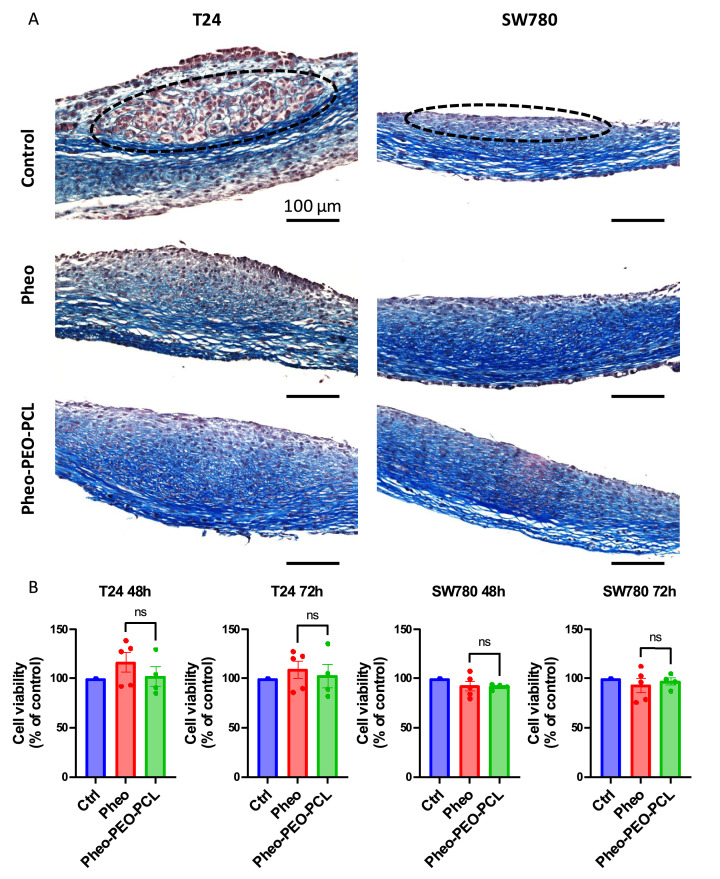
**Proof of concept of photodynamic therapy efficacy in a complex 3D engineered bladder tumor model.** A. Histological cross-sections of the vesical reconstructed tissues stained with Masson’s trichrome, 72 hours after PDT with 3 µM of either free pheophorbide a (Pheo) or pheophorbide *a* encapsulated in poly (ethylene oxide)-block-poly (ε-caprolactone) PEO-PCL micelles (Pheo–PEO-PCL). Cells appear in red, and stromal collagens in blue. Tumor cells are outlined with black dotted lines. Representative images are shown. **B.** Tissue viability assessed using the PrestoBlue assay at 48- and 72-hours post-PDT on engineered bladder substitutes implanted with T24 or SW780 spheroids. Results are expressed as a percentage of the untreated control at each timepoint ± SEM.

To enhance the translational relevance of *in vitro* findings, we explored PDT efficacy in a complex tissue-engineered model of human BC. This model incorporates a multilayered stroma composed of bladder fibroblasts and rich in endogenous extracellular matrix, overlaid with a differentiated urothelium, and has been previously shown to support RT4 and T24 tumor spheroid growth [30]. Using this approach, five pre-formed spheroids of either T24 or SW780 cells were deposited on the reconstructed tissues once a basement membrane was secreted between the stromal and epithelial compartments. T24 spheroids remained visible as distinct nodules seven days after seeding (Figure S1), whereas SW780 cells failed to maintain identifiable tumor masses in this structured environment and were not included in the figure. This behavior contrasts with their respective growth in standard ultra-low binding 96-well plates, where SW780 spheroids were significantly larger (275,210 ± 2234 µm² *vs.*44,904 ± 785 µm² for T24). These observations suggest that the presence of a rich stromal and epithelial microenvironment may differentially constrain the growth of low-grade tumor cells compared to high-grade T24 cells. Preliminary histological analyses performed 72 h after PDT with 3 µM pheophorbide *a* (either free or encapsulated in PEO-PCL micelles) revealed a marked reduction in visible tumor structures in the treated tissues compared to untreated controls (Fig. 5A). T24 masses were clearly detectable in control sections and appeared reduced or barely visible following PDT. For SW780, cells were present in the control tissues whereas none were visible in PDT-treated conditions. While these results remain preliminary and qualitative, they nonetheless indicate that PDT can effectively reduce tumor mass in this tissue-engineered BC model. To complement these morphological findings, a preliminary viability analysis was conducted at 48 and 72 h post-treatment (Figure 5B). Due to the limited number of replicates in the untreated control group (*n* = 1), no formal statistical comparison could be made to assess the overall efficacy of PDT. However, a reliable comparison between treatment conditions (free Pheo, *n* = 5; Pheo–PEO-PCL, *n* = 4) confirmed the absence of significant differences in tissue viability between the two formulation, whatever the cell line and the timing post-treatment. While preliminary, these results demonstrate the technical feasibility of applying PDT within this complex 3D model, rather than providing conclusive evidence of treatment efficacy. We acknowledge that the 3D tumor reconstructed-tissue assays were performed with a limited number of biological replicates, which constrains the statistical power and prevents drawing firm conclusions about PDT efficacy at this stage. As such, these experiments should be considered preliminary and primarily intended to assess the technical feasibility of applying PDT in this complex tissue-engineered model. Nevertheless, this approach represents an important step beyond conventional 2D and spheroid models, and lays the groundwork for future, fully powered experiments to quantitatively assess therapeutic responses in a physiologically relevant BC environment. This approach is in line with growing efforts to develop physiologically relevant 3D models that recapitulate the complexity of the tumor microenvironment. Standard monoculture spheroids, while useful, lack the intricate cellular diversity, extracellular matrix composition, and mechanical cues that characterize native tissues [43]. Strategies such as mixed-cell spheroids combining primary bladder fibroblasts or smooth muscle cells with BC cell lines to better mimic epithelial-stromal interactions [44], patient-derived tumoroids preserving intratumoral heterogeneity [45], or advanced engineered tissues with organized stroma and differentiated urothelium [30] are promising avenues for improving the predictive value of *in vitro* photodynamic therapy screening.

Given that photodynamic diagnosis (PDD) with hexaminolevulinate and blue-light cystoscopy is already widely adopted in BC management, the clinical infrastructure for intravesical photosensitizer delivery and light activation is well established. Recent advances have renewed interest in photodynamic therapy (PDT) for non-muscle-invasive bladder cancer (NMIBC), particularly in BCG-unresponsive cases. A meta-analysis of 28 clinical studies reported a complete response rate of about 68 %, with 12-month recurrence rates between 56 % and 84 % [12]. Adverse effects were mostly mild and transient, and the possibility of outpatient administration with bladder preservation makes PDT an attractive, patient-centered option. Building on this framework, PDT using pheophorbide *a* encapsulated in polymeric micelles can enhance intracellular delivery without major changes to existing protocols. This approach aligns with recent nanomedicine strategies, including pyropheophorbide a-loaded lipid nanoparticles (PPBC LNPs), which showed deep tumor penetration and image-guided therapeutic potential in preclinical bladder cancer models [46]. Clinically, the BRIGHT study demonstrates that photodynamic diagnosis (PDD)-guided transurethral resection of bladder tumor significantly reduced short-term intravesical recurrence in high-risk NMIBC patients [47]. These findings highlight the translational potential of integrating nanocarrier-mediated PDT into existing diagnostic and therapeutic workflows in bladder cancer.

## Conclusions

Results support the potential of photodynamic therapy (PDT) for bladder cancers, particularly when combined with polymer-based drug delivery. Encapsulating the pheophorbide a photosensitizer in self-assembled PEO-PCL micelles significantly enhanced cellular penetration and anticancer efficacy in 2D monolayers and 3D spheroid models. This formulation enabled determination of IC₅₀ values in monolayers, which was not possible with free Pheo, and improved PDT outcomes at later time points in 3D spheroids. These findings highlight the importance of nanocarrier-assisted delivery to overcome the limited bioavailability and tissue penetration of free photosensitizers. Clinically, this strategy is particularly appealing because existing photodynamic diagnosis (PDD) infrastructure, including blue-light cystoscopy and FDA-/EMA-approved photosensitizers, can be repurposed for PDT. The combination of improved drug delivery and current clinical devices could accelerate integration of PDT into bladder-sparing treatment protocols, offering a minimally invasive, localized, and potentially cost-effective therapeutic option.

## Funding

This work was supported by Conseil franco-québécois de coopération universitaire (CFQCU) Samuel de Champlain grant (2021/011) to LG and FB. FB is a tier 2 Canada Research Chair in Tumor Mechanobiology and Cellular Mechanoregulation. SB was a recipient of the Canadian Urological Association Scholarship Fund (CUASF) and a grant by CUASF-Bladder Cancer Canada. This work was supported by the Fonds de recherche du Québec-Santé (FRQS) through the research centre grant for the CHU de Québec-Université Laval Research Center (reference: 30641).

## Consent to publish declaration

Not applicable.

## Ethics and consent to participate declarations

Not applicable.

## CRediT authorship contribution statement

**Maxime Labroy:** Visualization, Methodology, Investigation. **Stéphane Chabaud:** Writing – review & editing, Project administration, Methodology, Formal analysis, Conceptualization. **Maud Durand:** Investigation. **Isabelle Fourquaux:** Visualization. **Stéphane Bolduc:** Writing – review & editing, Project administration, Funding acquisition. **François Bordeleau:** Writing – review & editing, Supervision, Investigation, Funding acquisition. **Laure Gibot:** Writing – review & editing, Writing – original draft, Visualization, Supervision, Project administration, Investigation, Funding acquisition, Formal analysis, Conceptualization.

## Declaration of competing interest

The authors declare the following financial interests/personal relationships which may be considered as potential competing interests: Gibot reports financial support was provided by Conseil franco-québécois de coopération universitaire (CFQCU). Bordeleau reports financial support was provided by Canada Research Chair in Tumor Mechanobiology and Cellular Mechanoregulation. Bolduc reports financial support was provided by Canadian Urological Association Scholarship Fund (CUASF). Bolduc reports financial support was provided by CUASF-Bladder Cancer Canada. If there are other authors, they declare that they have no known competing financial interests or personal relationships that could have appeared to influence the work reported in this paper.

## Appendix Supplementary materials

Image, video 1 dummy alt text

Image, video 2 dummy alt text

Image, application 3

## References

[1] Zhang Y., Rumgay H., Li M., Yu H., Pan H., Ni J. (2023). The global landscape of bladder cancer incidence and mortality in 2020 and projections to 2040. J. Glob. Health.

[2] Olislagers M., de Jong F.C., Rutten V.C., Boormans J.L., Mahmoudi T., Zuiverloon T.C.M. (2025). Molecular biomarkers of progression in non-muscle-invasive bladder cancer — Beyond conventional risk stratification. Nat. Rev. Urol..

[3] St-Laurent M.-P., Bochner B., Catto J., Davies B.J., Fankhauser C.D., Garg T. (2025). Increasing life expectancy in patients with genitourinary malignancies: impact of treatment burden on disease management and quality of life. Eur. Urol..

[4] D’Andrea D., Mostafid H., Gontero P., Shariat S., Kamat A., Masson-Lecomte A. (2025). Unmet need in non–muscle-invasive Bladder Cancer failing Bacillus Calmette-Guérin therapy: a systematic review and cost-effectiveness analyses from the International Bladder Cancer Group. European Urol. Oncol..

[5] Gontero P., Birtle A., Capoun O., Compérat E., Dominguez-Escrig J.L., Liedberg F. (2024). European Association of Urology Guidelines on non–muscle-invasive bladder cancer (TaT1 and Carcinoma *In Situ*)—A summary of the 2024 Guidelines update. Eur. Urol..

[6] Babjuk M., Burger M., Compérat E., Gontero P., Mostafid A.H., Palou J. (2020). http://uroweb.org/guideline/non-muscle-invasive-bladder-cancer/.

[7] Arora A., Godse S., Pal M., Misra A., Sepuri R.T., Mallikarjun N.T. (2025). Real-world oncological and toxicity outcomes with the Moscow strain of intravesical BCG for non-muscle invasive bladder cancer—Implications for global shortage. BJUI. Compass..

[8] Bertrand A., Roumeguere T., Vandamme J., Spinoit A.-F., Herve F., Assenmacher C. (2025). National Belgian survey on managing non-muscle invasive bladder cancer during Mitomycin and bacillus Calmette-Guerin shortage. French J. Urol..

[9] Gurbani C.M., Chong Y.-L., Choo Z.W., Chia D., Chia P.L., Vong E. (2025). Emerging bladder-sparing treatments for high risk non-muscle invasive bladder cancer. Bladder. Cancer.

[10] Kausch I., Sommerauer M., Montorsi F., Stenzl A., Jacqmin D., Jichlinski P. (2010). Photodynamic diagnosis in non–Muscle-invasive bladder cancer: a systematic review and cumulative analysis of prospective studies. Eur. Urol..

[11] Benson R.C., Kinsey J.H., Cortese D.A., Farrow G.M., Utz D.C (1983). Treatment of transitional cell carcinoma of the bladder with hematoporphyrin derivative phototherapy. Journal of Urology. WoltersKluwer;.

[12] Li H., Long G., Tian J. (2023). Efficacy and safety of photodynamic therapy for non–muscle-invasive bladder cancer: a systematic review and meta-analysis. Front Oncol [Internet].

[13] Kochergin M., Fahmy O., Asimakopoulos A., Theil G., Zietz K., Bialek J. (2024). Photodynamic therapy: current trends and potential future role in the treatment of bladder cancer. Int. J. Mol. Sci..

[14] Saide A., Lauritano C., Ianora A. (2020). Pheophorbide a: state of the art. Mar. Drugs.

[15] Chountoulesi M., Selianitis D., Pispas S., Pippa N. (2023). Recent advances on PEO-PCL block and graft copolymers as nanocarriers for drug delivery applications. Materials. (Basel).

[16] Gibot L., Demazeau M., Pimienta V., Mingotaud A.-F., Vicendo P., Collin F. (2020). Role of polymer micelles in the delivery of photodynamic therapy agent to liposomes and cells. Cancers. (Basel).

[17] Gibot L., Lemelle A., Till U., Moukarzel B., Mingotaud A.-F., Pimienta V. (2014). Polymeric micelles encapsulating photosensitizer: structure/photodynamic therapy efficiency relation. Biomacromolecules..

[18] Till U., Gibot L., Mingotaud A.-F., Ehrhart J., Wasungu L., Mingotaud C. (2016). Drug release by direct jump from poly(ethylene-glycol-b-ε-caprolactone) nano-vector to cell membrane. Molecules..

[19] Till U., Gibot L., Mingotaud C., Vicendo P., Rols M.-P., Gaucher M. (2016). Self-assembled polymeric vectors mixtures: characterization of the polymorphism and existence of synergistic effects in photodynamic therapy. Nanotechnology..

[20] Brival R., Ghafari N., Mingotaud A.-F., Fourquaux I., Gilard V., Collin F. (2024). Encapsulation of photosensitizer worsen cell responses after photodynamic therapy protocol and polymer micelles act as biomodulators on their own. Int. J. Pharm..

[21] Zheng X., Lordon B., Mingotaud A., Vicendo P., Brival R., Fourquaux I. (2023). Terahertz spectroscopy sheds light on real-time exchange kinetics occurring through plasma membrane during photodynamic therapy treatment. Adv. Sci..

[22] Sarigul N., Korkmaz F., Kurultak İ. (2019). A new artificial urine protocol to better imitate Human urine. Sci. Rep..

[23] Till U., Gibot L., Vicendo P., Rols M.-P., Gaucher M., Violleau F. (2016). Crosslinked polymeric self-assemblies as an efficient strategy for photodynamic therapy on a 3D cell culture. RSC. Adv..

[24] Oudin A., Chauvin J., Gibot L., Rols M.-P., Balor S., Goudounèche D. (2019). Amphiphilic polymers based on polyoxazoline as relevant nanovectors for photodynamic therapy. J. Mater. Chem. B. Royal Society of Chem..

[25] Gibot L., Rols M.-P. (2013). 3D spheroids’ sensitivity to electric field pulses depends on their size. J. Membr. Biol..

[26] Frandsen S.K., Gibot L., Madi M., Gehl J., Rols M.-P. (2015). Calcium electroporation: evidence for differential effects in normal and malignant cell lines, evaluated in a 3D spheroid model. PLoS. One.

[27] Gouarderes S., Ober C., Doumard L., Dandurand J., Vicendo P., Fourquaux I. (2022). Pulsed electric fields induce extracellular matrix remodeling through matrix metalloproteinases activation and decreased collagen production. J. Invest. Dermatol..

[28] Athanasiou K.A., Eswaramoorthy R., Hadidi P., Hu J.C. (2013). Self-organization and the Self-assembling process in tissue engineering. Annu Rev. Biomed. Eng..

[29] Roy V., Magne B., Vaillancourt-Audet M., Blais M., Chabaud S., Grammond E. (2020). Human organ-specific 3D cancer models produced by the stromal self-assembly method of tissue engineering for the study of solid tumors. Biomed. Res. Int..

[30] Goulet C.R., Bernard G., Chabaud S., Couture A., Langlois A., Neveu B. (2017). Tissue-engineered human 3D model of bladder cancer for invasion study and drug discovery. Biomaterials.

[31] Brownell D., Carignan L., Alavi R., Caneparo C., Labroy M., Galbraith T. (2025). Impact of the use of 2-phospho-L ascorbic acid in the production of engineered stromal tissue for regenerative medicine. Cells.

[32] Ma J., Guo C., Tang Y., Chen L., Bahadur P., Liu H. (2007). Interaction of urea with pluronic block copolymers by 1H NMR spectroscopy. J. Phys. Chem. B.

[33] Vasyutin I., Zerihun L., Ivan C., Atala A. (2019). Bladder organoids and spheroids: potential tools for normal and diseased tissue modelling. Anticancer Res..

[34] Gildea J.J., Golden W.L., Harding M.A., Theodorescu D. (2000). Genetic and phenotypic changes associated with the acquisition of tumorigenicity in human bladder cancer. Genes, Chromosomes and Cancer..

[35] Roelants M., Cleynenbreugel B.V., Lerut E., Poppel H.V., de Witte PAM (2011). Human serum albumin as key mediator of the differential accumulation of hypericin in normal urothelial cell spheroids versusurothelial cell carcinoma spheroids. Photochem. Photobiol. Sci..

[36] Fam S.Y., Chee C.F., Yong C.Y., Ho K.L., Mariatulqabtiah A.R., Tan W.S. (2020). Stealth coating of nanoparticles in drug-delivery systems. Nanomaterials. (Basel).

[37] Xiao Z., Hansen C.B., Allen T.M., Miller G.G., Moore R.B. (2005). Distribution of photosensitizers in bladder cancer spheroids: implications for intravesical instillation of photosensitizers for photodynamic therapy of bladder cancer. J. Pharm. Pharm. Sci..

[38] Vandepitte J., Roelants M., Van Cleynenbreugel B., Hettinger K., Lerut E., Van Poppel H. (2011). Biodistribution and photodynamic effects of polyvinylpyrrolidone-hypericin using multicellular spheroids composed of normal human urothelial and T24 transitional cell carcinoma cells. J. Biomed. Opt..

[39] Kataoka K., Harada A., Nagasaki Y. (2012). Block copolymer micelles for drug delivery: design, characterization and biological significance. Adv. Drug Deliv. Rev..

[40] Kerdous R., Sureau F., Bour A., Bonneau S. (2015). Release kinetics of an amphiphilic photosensitizer by block-polymer nanoparticles. Int. J. Pharm..

[41] Savić R., Luo L., Eisenberg A., Maysinger D. (2003). Micellar nanocontainers distribute to defined cytoplasmic organelles. Science.

[42] Zhuo Z., Song Z., Ma Z., Zhang Y., Xu G., Chen G. (2019). Chlorophyllin e6‑mediated photodynamic therapy inhibits proliferation and induces apoptosis in human bladder cancer cells. Oncol. Rep..

[43] Bordeleau F., Brownell D., Chabaud S., Huot M.-E., Bolduc S. (2023). Recreating heterogeneity of bladder cancer microenvironment to study its recurrences and progression. Stem Cell Investig..

[44] Berndt-Paetz M., Han S., Weimann A., Reinhold A., Nürnberger S., Neuhaus J. (2023). Cell line-based Human bladder organoids with bladder-like self-organization—A new standardized approach in bladder cancer research. Biomedicines..

[45] Yang R., Wang S., Li Z., Yin C., Huang W., Huang W. (2025). Patient-derived organoid co-culture systems as next-generation models for bladder cancer stem cell research. Cancer Lett..

[46] Tang M., Mahri S., Shiau Y.-P., Mukarrama T., Villa R., Zong Q. (2025). Multifunctional and scalable nanoparticles for bimodal image-guided phototherapy in bladder cancer treatment. Nanomicro Lett..

[47] Kawai T., Matsuyama H., Kobayashi K., Ikeda A., Miyake M., Nishimoto K. (2024). Photodynamic diagnosis-assisted transurethral resection of bladder tumor for high-risk non-muscle invasive bladder cancer improves intravesical recurrence-free survival (BRIGHT study). Int. J. Urol..

