# Supplementary material for: Improving photodynamic therapy efficacy in bladder cancer using polymer micelle-encapsulated pheophorbide a
Source: Transl Oncol. 2026 Feb 6;65:102687. doi: 10.1016/j.tranon.2026.102687 (PMC12903199; doi:10.1016/j.tranon.2026.102687)
Supplement: Supplementary file 3 [file mmc3.pptx]

## Slide 1
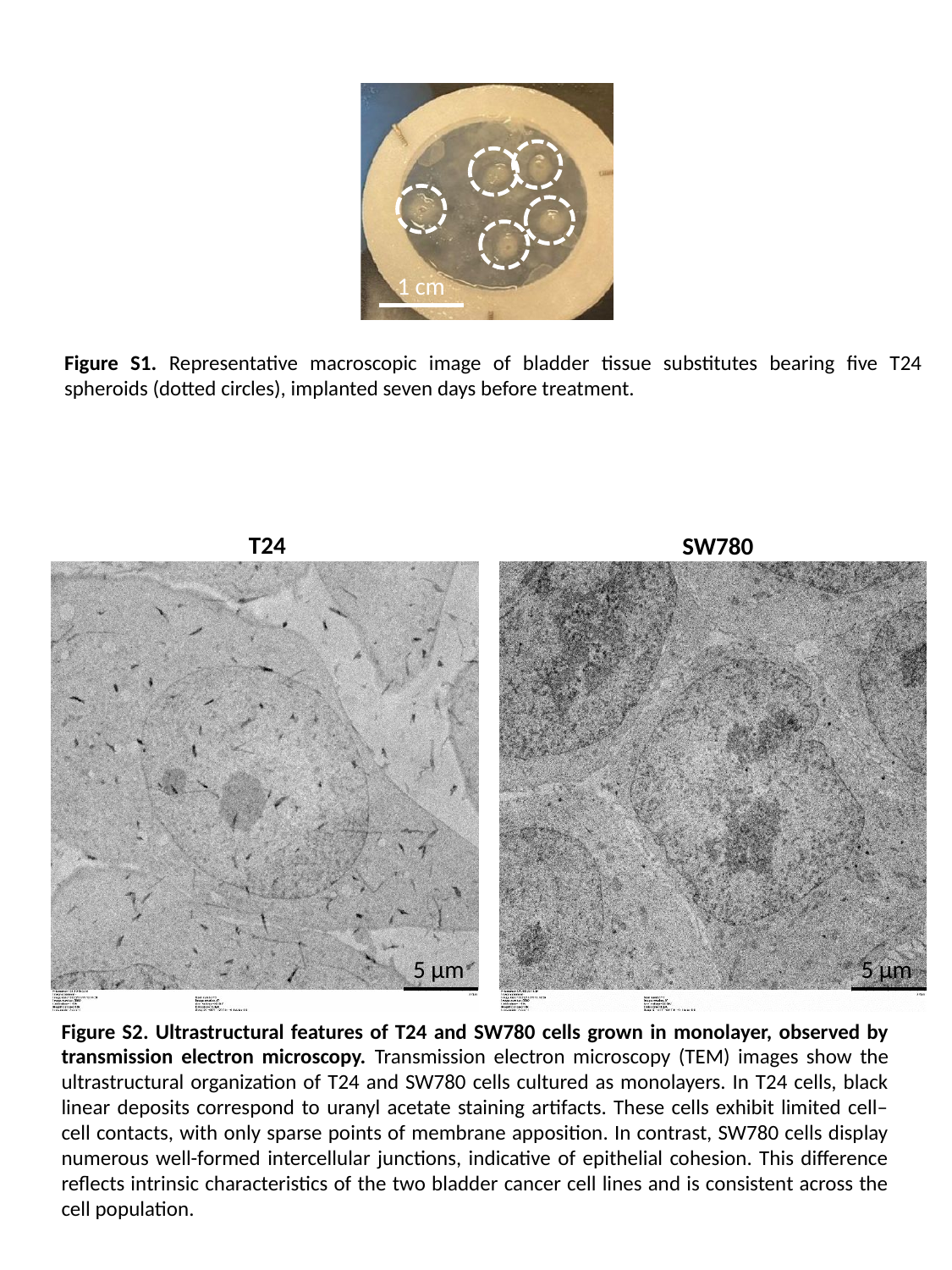

1 cm
Figure S1. Representative macroscopic image of bladder tissue substitutes bearing five T24 spheroids (dotted circles), implanted seven days before treatment.
T24
SW780
5 µm
5 µm
Figure S2. Ultrastructural features of T24 and SW780 cells grown in monolayer, observed by transmission electron microscopy. Transmission electron microscopy (TEM) images show the ultrastructural organization of T24 and SW780 cells cultured as monolayers. In T24 cells, black linear deposits correspond to uranyl acetate staining artifacts. These cells exhibit limited cell–cell contacts, with only sparse points of membrane apposition. In contrast, SW780 cells display numerous well-formed intercellular junctions, indicative of epithelial cohesion. This difference reflects intrinsic characteristics of the two bladder cancer cell lines and is consistent across the cell population.

## Slide 2
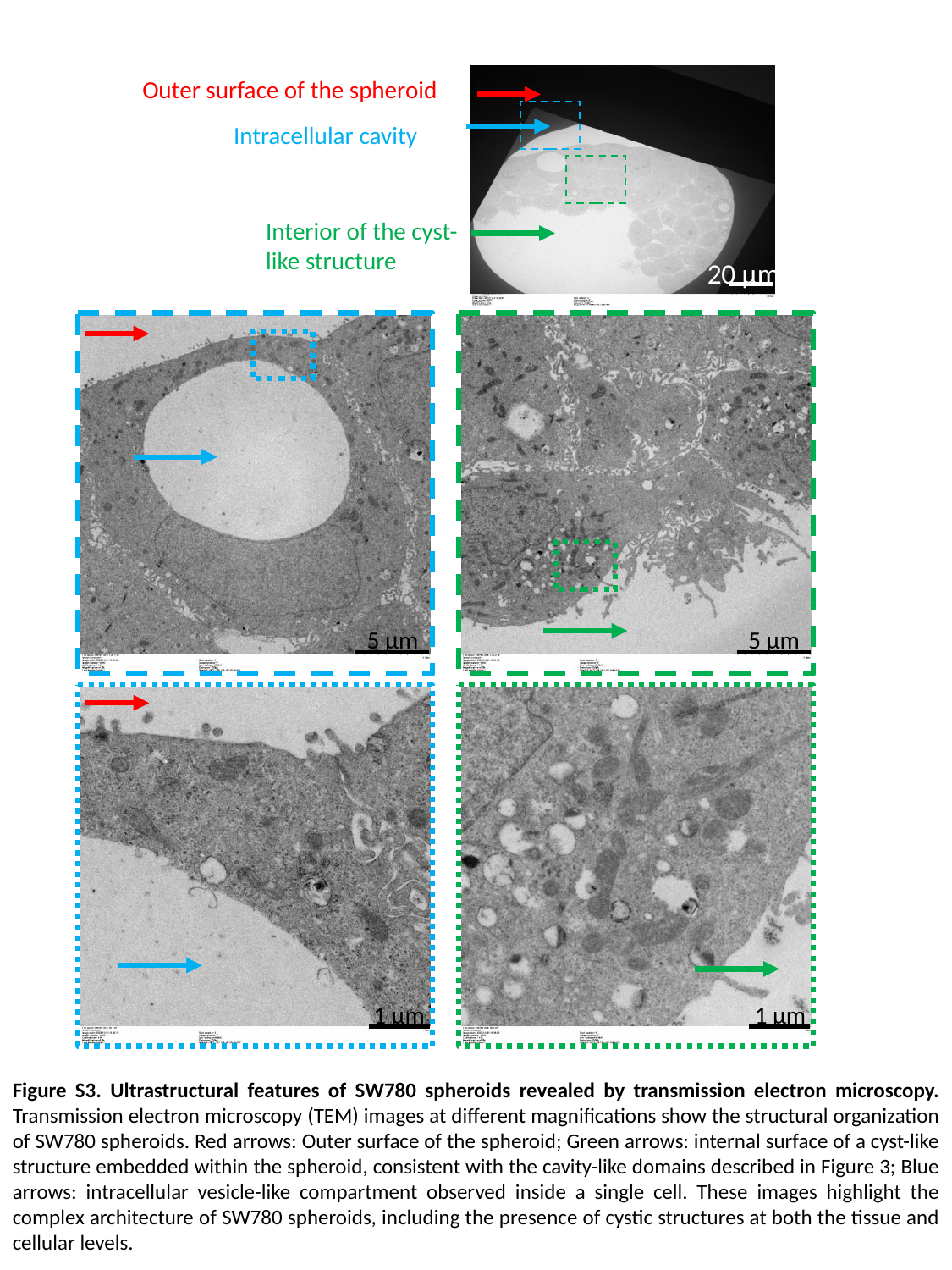

Outer surface of the spheroid
Intracellular cavity
Interior of the cyst-like structure
20 µm
5 µm
5 µm
1 µm
1 µm
Figure S3. Ultrastructural features of SW780 spheroids revealed by transmission electron microscopy. Transmission electron microscopy (TEM) images at different magnifications show the structural organization of SW780 spheroids. Red arrows: Outer surface of the spheroid; Green arrows: internal surface of a cyst-like structure embedded within the spheroid, consistent with the cavity-like domains described in Figure 3; Blue arrows: intracellular vesicle-like compartment observed inside a single cell. These images highlight the complex architecture of SW780 spheroids, including the presence of cystic structures at both the tissue and cellular levels.

## Slide 3
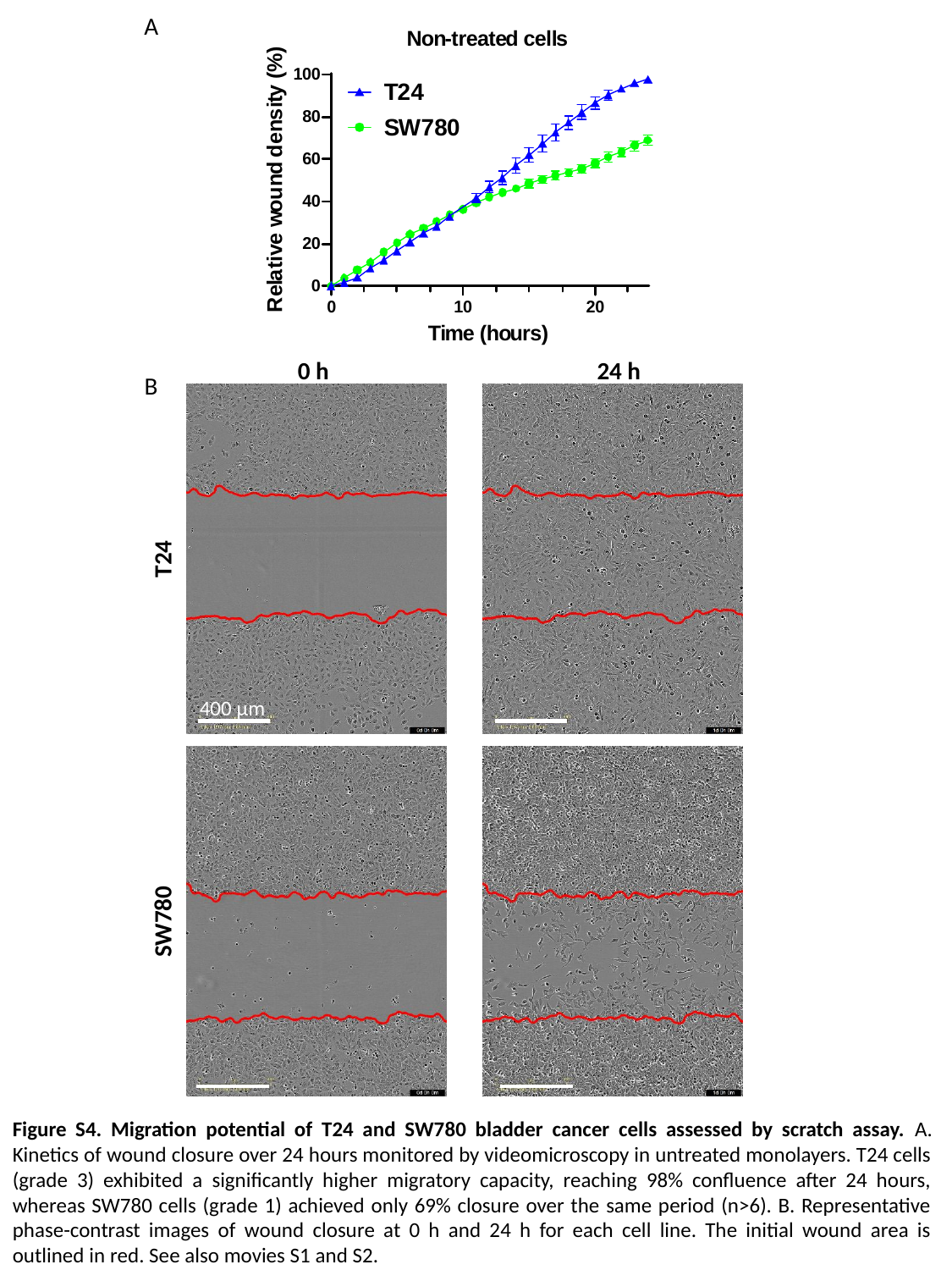

A
0 h
24 h
B
T24
400 µm
SW780
Figure S4. Migration potential of T24 and SW780 bladder cancer cells assessed by scratch assay. A. Kinetics of wound closure over 24 hours monitored by videomicroscopy in untreated monolayers. T24 cells (grade 3) exhibited a significantly higher migratory capacity, reaching 98% confluence after 24 hours, whereas SW780 cells (grade 1) achieved only 69% closure over the same period (n>6). B. Representative phase-contrast images of wound closure at 0 h and 24 h for each cell line. The initial wound area is outlined in red. See also movies S1 and S2.
